# The Impact of Cangrelor in the UK for the Treatment of STEMI Patients with Gastric Absorption Issues Undergoing Percutaneous Coronary Intervention

**DOI:** 10.3390/jcm14217564

**Published:** 2025-10-25

**Authors:** Bhavik Modi, Rob Cain, Richard Stork, Gina Tarpey, Alessia Colucciello, Danielle Olivier, Caroline Barwood, Will Wright, Rory McAtamney

**Affiliations:** 1 Glenfield Hospital, University Hospitals of Leicester NHS Trust, Leicester LE3 9QP, UK; bhavik.modi@uhl-tr.nhs.uk; 2 Chiesi Ltd., Manchester M22 5LG, UK; r.cain@chiesi.com (R.C.); g.tarpey@chiesi.com (G.T.); 3 Chiesi Farmaceutici S.p.A., Via Palermo, 26/A, 43122 Parma, Italy; a.colucciello@chiesi.com; 4 FIECON Ltd., London EC1R 3AW, UK; dani.olivier@fiecon.com (D.O.); caroline.barwood@fiecon.com (C.B.); will.wright@fiecon.com (W.W.); rory.mcatamney@fiecon.com (R.M.)

**Keywords:** glycoprotein inhibitor, P2Y12 inhibitor, cardiovascular disease, gastric absorption, antiplatelet therapy

## Abstract

**Background/Objectives**: Patients that undergo percutaneous coronary intervention (PCI) require effective antiplatelet therapies to minimize the risk of thrombotic cardiovascular events. Oral P2Y12 inhibitors are often utilized, however co-administered opioids may lead to gastric absorption issues in these patients, affecting the efficacy of oral inhibitors. Cangrelor is an intravenous, direct-acting, reversible P2Y12 inhibitor that could be explored as a potential treatment option for patients with gastric absorption issues during ST-elevation myocardial infarction. The objective was to estimate the UK budget impact of introducing cangrelor for ST-elevation myocardial infarction (STEMI) patients with gastric absorption issues undergoing PCI. **Methods**: A budget impact model was developed to calculate the impact of introducing cangrelor to treat STEMI patients with gastric absorption issues undergoing PCI, to the UK National Health Service and personal social services, over 5 years. Oral P2Y12 inhibitors (clopidogrel, prasugrel, and ticagrelor), glycoprotein IIb/IIIa inhibitors (eptifibatide and tirofiban), and aspirin and heparin alone were included as base case comparators. Cangrelor uptake ranged from 10% to 30% in years 1–5. The cangrelor-eligible population was estimated at 10,903 patients per year. **Results**: Over 5 years, cangrelor leads to a small cost saving (0.29%), varying from −GBP 261,989 in year 1 to GBP 174,778 in year 5. The introduction of cangrelor is estimated to lead to 314 fewer hospital days and 190 clinical events avoided over 5 years. **Conclusions**: Introducing cangrelor to STEMI patients with gastric absorption issues undergoing PCI in the UK is estimated to generate a small cost saving and reduced length of stay for some patients.

## 1. Introduction

Cardiovascular diseases (CVDs) encompass a broad array of conditions affecting around 7.6 million people in the UK, accounting for 170,000 deaths a year [1]. As a result, CVD has a substantial economic burden, with annual healthcare costs estimated at GBP 10 billion [1].

ST-elevation myocardial infarction (STEMI) is one of the most severe categories of CVD, with 3 in 10 events proving fatal and accounting for around 100,000 hospital admissions each [1]. Percutaneous coronary intervention (PCI) is a common non-surgical invasive procedure performed on STEMI patients, with the goal of minimizing the risk of morbidity or death by relieving the narrowing or occlusion of the coronary artery [2]. According to recent data from the British Cardiovascular Intervention Society (BCIS), around 100,000 PCI procedures are performed annually on patients in the UK, with approximately 1 in 5 performed on STEMI patients [3,4].

When undergoing PCI procedures, immediate and effective platelet inhibition is imperative to minimize the risk of ischemic events, particularly stent thrombosis [3]. Platelet inhibition can be achieved using glycoprotein IIb/IIIa receptor inhibitors (GPIs), such as eptifibatide or tirofiban, or oral P2Y12 inhibitors, such as clopidogrel, prasugrel or ticagrelor [5,6]. While GPIs are safe and effective, they are typically reserved for use in high-risk PCI in patients who have not been pre-treated with oral P2Y12 inhibitors, or for use as bailout [6,7]. While oral P2Y12 inhibitors are effective, their benefits are frequently limited by their delayed onset of action, requiring 4–6 hours to achieve total platelet inhibition; therefore, oral P2Y12 inhibitors may be unsuitable for STEMI patients with gastric absorption issues [8].

Gastric absorption issues among STEMI patients are common, often because of opioid treatment. Opioids, such as morphine or fentanyl, are often recommended for pain relief in STEMI patients undergoing PCI but are associated with side effects of vomiting and delayed gastric emptying, resulting in absorption delays of platelet inhibitors and hindering drug efficacy [5,9]. Furthermore, opioids have been associated with enhanced platelet reactivity and reduced spontaneous reperfusion, resulting in larger infarct size and delayed gastric absorption [7]. Given that oral P2Y12 inhibitors may not be effective in STEMI patients with gastric absorption issues, intravenous (IV) P2Y12 inhibitors may be required.

Cangrelor is an IV, fast-acting, rapidly reversible platelet adenosine diphosphate (ADP) P2Y12 inhibitor that inhibits platelet function [3]. The effects of cangrelor are reversible, with normal platelet function restored within one hour after discontinuation [10]. When used alongside acetylsalicylic acid, cangrelor is indicated for reducing the risk of thrombotic cardiovascular events in adult patients with coronary artery disease undergoing PCI, who have not received an oral P2Y12 inhibitor before the PCI procedure, and in whom oral therapy with P2Y12 inhibitors is not feasible or desirable [11]. It is referenced as a treatment option in the 2023 European Society of Cardiology (ESC) guidelines for the management of acute coronary syndromes [6].

The CHAMPION program (CHAMPION-PCI, CHAMPION-PLATFORM, and CHAMPION-PHOENIX) has demonstrated that, compared with clopidogrel, cangrelor significantly reduces the rate of ischemic events, including stent thrombosis during PCI, with no significant increases in severe bleeding [3,12,13].

While the CHAMPION trials have demonstrated the efficacy and safety of cangrelor in patients undergoing PCI, including STEMI patients with gastric absorption issues, clinical expert opinion has validated the idea that cangrelor is generally only used in UK centres for the out-of-hospital cardiac arrest (OHCA) population. Given the potential value of cangrelor in STEMI patients with gastric absorption issues, we conducted an economic model to calculate the 5-year budget impact of introducing cangrelor to UK hospitals for STEMI patients with gastric absorption issues undergoing PCI.

## 2. Materials and Methods

### 2.1. Overview

An economic model was built to calculate the 5-year budget impact to the UK National Health Service (NHS) and personal social services (PSS) of the introduction of cangrelor for the management of STEMI patients with gastric absorption issues who require PCI. The model considers two hypothetical scenarios. One scenario considers a market without cangrelor, with patients receiving either GPIs (eptifibatide or tirofiban), oral P2Y12 inhibitors (clopidogrel, prasugrel, or ticagrelor), or aspirin and heparin alone in combination. The second scenario considers the inclusion of cangrelor as an additional option alongside GPIs, oral P2Y12 inhibitors, or aspirin-heparin.

### 2.2. Inputs and Data Sources

A targeted literature review was conducted to identify the most relevant published data to ensure the model accurately reflects the usual clinical practice in the UK. Assumptions were made in the absence of data, but all key inputs and assumptions were validated by a UK consultant interventional cardiologist.

### 2.3. Target Population

Hospital Episode Statistics (HES) is a database containing information on hospital services across England, including hospital admissions, length of stay for patients, and outpatient appointments. HES data were used to estimate the population of STEMI patients with gastric absorption issues who are eligible to receive cangrelor [14]. The data showed that, in 2023, 94,103 PCIs were carried out in the UK. Of these PCI procedures, 21,318 were performed on STEMI patients. Through a review of published literature and consultation with a UK consultant interventional cardiologist, the proportion of STEMI patients with gastric absorption issues was estimated to be 50% (10,903 patients) [5,9,15,16,17]. This estimate was deemed appropriate for a UK healthcare setting but may vary across healthcare systems. The impact of this assumption was explored through scenario analysis. The eligible population was assumed to remain stable year-on-year, driven by consistent annual PCI volumes in the UK. The number of procedures remained relatively stable over the past few years in the UK: 100,112 in 2019/20, 90,708 in 2020/21 (impacted by the COVID-19 pandemic), 97,765 in 2021/22, and 94,103 in 2022/23 [18,19,20,21]. Expert clinical opinion was used to inform estimates of the current market share of antiplatelet treatments among patients, as well as to project uptake rates for cangrelor in the UK over the subsequent 5-year period, highlighted in Table 1.

### 2.4. Efficacy and Safety Data

Efficacy and safety data for cangrelor and comparators were included in the budget impact model (BIM) to estimate resource use and costs associated with clinical events. Given the scarcity of efficacy data for the combination of aspirin and heparin, it was assumed that their performance would be comparable to that of clopidogrel. The effects of this assumption were explored through scenario analysis, with a reduction in the efficacy of aspirin-heparin showing a minimal budget impact. Efficacy data for prasugrel and ticagrelor were sourced from a network meta-analysis of 15 randomized controlled trials by Westman et al. [22]. Efficacy data for eptifibatide and tirofiban were derived from a pooled analysis of the CHAMPION trials, focusing on patients who received standard glycoprotein IIb/IIIa inhibitors [23]. Where possible, odds ratios stated in Westman et al. were utilized to adjust efficacy and safety data to be specific to STEMI patients [22]. See Table 2 for a comprehensive summary of the efficacy and safety data used in the model.

### 2.5. Length of Stay Data

Length of stay (LOS) data for patients undergoing PCI using comparator therapies were utilized to assess the impact of cangrelor on time spent in the hospital and were sourced from HES data [14]. Post-operative stay for comparators was calculated as the mean LOS for non-elective PCI minus the mean pre-operative LOS. Post-operative LOS data for cangrelor were calculated by applying the percentage reduction in LOS from a US-based study of cangrelor versus GPIs during PCI (GPI LOS: 4.3 days, cangrelor: 3.5 days, 19% reduction) [25]. Whilst post-operative LOS data in the UK were limited, they were deemed the best available data for assessing the budget impact of cangrelor. LOS for UK patients treated with GPIs was estimated at 3.10 days based on HES data [14]. Therefore, a 19% reduction was then applied to the GPI LOS to give an LOS for PCI for cangrelor-treated patients of 2.52 days. LOS for aspirin-heparin and oral P2Y12 inhibitors were conservatively assumed equal to cangrelor, assuming no LOS benefit versus this comparator.

### 2.6. GPI Bailout Use

Bailout GPIs are defined as the use of GPI when the PCI operator has not intended to use GPI from the outset but considers that clinical or angiographic features (such as worsening or persistent thrombus burden) have changed during the procedure, such that there may be a benefit to giving the patient GPI [26]. Bailout GPI use was conservatively assumed to decrease from 10% to 2.2% upon the introduction of cangrelor due to its ability to reduce thrombotic cardiovascular events when compared with oral P2Y12 inhibitors, based on Thim et al., 2023 and confirmed by clinical expert opinion [27].

### 2.7. Cost Data

Treatment costs were sourced from the British National Formulary (BNF). The cost of an additional stay in hospital was sourced from the NHS reference costs and clinical event costs were sourced from existing published literature. The costs were updated to 2022/23 GBP based on the Unit Costs of Health and Social Care 2023 Manual and costs remain undiscounted from year 1 onwards based on the National Institute for Health and Care Excellence (NICE) and The Professional Society for Health Economics and Outcomes Research (ISPOR) budget impact analysis guidelines principles of good practice [28,29,30]. A complete summary of cost data used in the model is summarized in Table 3.

## 3. Results

The base case BIM shows that the total number of STEMI patients with gastric absorption issues eligible for cangrelor treatment reaches 54,513 over 5 years. Over the 5-year time horizon, a total of 10,903 patients is expected to receive treatment with cangrelor, with an average of 2181 patients treated per year. Over 5 years, the total costs incurred by the NHS and PSS in the scenario without cangrelor are GBP 74,948,153, whereas the total costs incurred by the NHS and PSS in the scenario with cangrelor are GBP 74,730,151. Therefore, in the base case, the introduction of cangrelor is expected to lead to a small cost saving of GBP 218,002 over 5 years (−0.29%), varying from −GBP 261,989 (−1.75%) in year 1, to GBP 174,788 (+1.17%) in year 5, driven by reduced bailout GPI usage. Over 5 years, approximately 314 hospital days and 190 clinical events are predicted to be avoided due to cangrelor use, including ST, MI, IDR, death and minor or major bleeds. While the absolute reductions may appear modest when considered across the total population of >54,000 patients, they translate to meaningful benefits at the patient level and represent a measurable reduction in serious adverse outcomes. Base-case budget impact results are available in Table 4.

### 3.1. Sensitivity Analysis

#### 3.1.1. One Way Sensitivity Analysis

**Figure 1:**
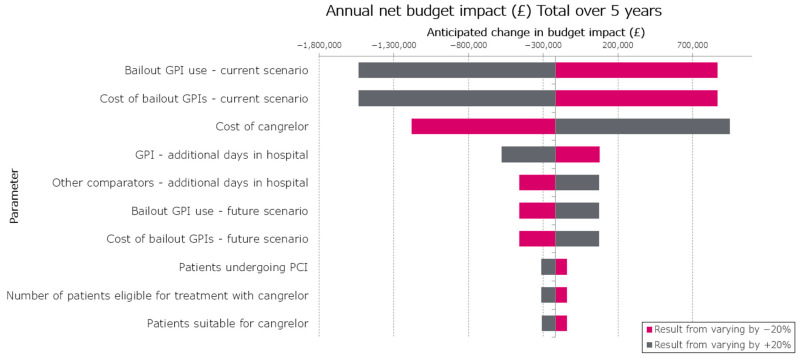
One-way sensitivity analysis results for total budget impact over 5 years (GBP). Abbreviations: GPI, glycoprotein inhibitor; PCI, percutaneous coronary intervention.

A one-way sensitivity analysis was conducted to assess the impact of different parameters on the budget impact results. Parameters were varied by ±20%, with additional consideration given to GPI cost, given uncertainty in patient weight and duration of infusion, assuming vial usage varies +/− one vial per patient in the OWSA. The 10 parameters with the largest effect on the budget impact were included in Figure 1.

The results of a one-way sensitivity analysis show that the model is most sensitive to the proportion of patients using a bailout GPI, cost of bailout GPIs and cost of cangrelor. Varying the proportion of patients using a bailout GPI by −20% led to a budget impact of GBP 868,552, whereas varying this parameter by +20% led to cost savings of GBP 1,537,205 (Table 5).

#### 3.1.2. Scenario Analysis

A scenario analysis was conducted assuming oral P2Y12s are not absorbed and therefore have equal efficacy to aspirin-heparin. This resulted in a small increase in the cost saving associated with cangrelor, from −GBP 218,002 in the base case (−0.29%) to −GBP 253,986 (−0.34%) over 5 years. A second scenario analysed the effects on the budget impact if cangrelor had a 19% reduction in hospital LOS compared with all comparators, not just GPIs (assuming any oral P2Y12 inhibitor is not absorbed, and that aspirin-heparin alone has minimal efficacy owing to lack of data). This scenario also resulted in an increase in cost saving with cangrelor, from −GBP 218,002 in the base case (−0.29%) to −GBP 3,195,858 (−3.56%) over 5 years.

A third scenario analysis was conducted to understand the impact of the assumption that 50% of STEMI patients experience gastric absorption issues, by assessing the budget impact effect if only 25% of STEMI patients experience gastric absorption issues. As the percentage of STEMI patients that experience gastric absorption issues due to opioid use decreases, so does the budget savings of cangrelor, from −GBP 218,002 in the base case (−0.29%) to −GBP 109,001 (−0.29%) over 5 years.

Finally, a scenario analysis was conducted that assumed a lower efficacy of aspirin-heparin than in the base case, where it was assumed equal to clopidogrel. Reducing the efficacy to 50% of the efficacy of clopidogrel has minimal budget impact, reducing the cost savings from −GBP 218,002 in the base case (−0.29%) to −GBP 212,939 (−0.28%) over 5 years.

## 4. Discussion

In patients with STEMI undergoing PCI, immediate and effective platelet inhibition is crucial to prevent thrombotic complications and reduce ischemic events [7,8]. Usually, platelet inhibition can be achieved through the use of oral P2Y12 inhibitors or intravenous GPIs [5,6]. While opioids such as morphine or fentanyl are often used for pain relief in STEMI patients undergoing PCI, they can cause side effects of vomiting and delayed gastric emptying, which in turn can delay absorption and potentially reduce the efficacy of oral P2Y12 inhibitors [5,9]. While GPIs are safe and effective, they are typically reserved for use in high-risk PCI in patients who have not been pre-treated with oral P2Y12 inhibitors, or for use as a bailout [6,7].

Cangrelor, a potent IV P2Y12 receptor inhibitor, provides rapid, effective and predictable onset and offset of platelet inhibition. Its reversible binding properties allow for quick reversal of its effects and facilitates timely interventions with reduced bleeding risks during PCI procedures [7].

While cangrelor is used in UK hospitals for the OHCA population, its use in STEMI patients with gastric absorption issues is limited. Therefore, a BIM was conducted to assess the economic impact of introducing cangrelor to UK hospital for STEMI patients with gastric absorption issues.

The results of the budget impact analysis demonstrate that introducing cangrelor leads to a small cost saving of GBP 218,002 over 5 years. Cost savings are driven by reduction in bailout GPI usage and reduced LOS in hospital. This assumes that bailout GPI usage falls significantly when IV cangrelor is used [27].

The results of this budget impact analysis are broadly consistent with other similar published analyses, which demonstrated that cangrelor introduction is associated with an affordable 3-year budget impact of EUR 115,000, EUR 500,000, EUR 1 million and EUR 1.1 million to the healthcare systems of Portugal [46], Germany [47], Spain [33], and Belgium [48], respectively, and reduced the number of patients treated with GPIs. The budget impact analyses conclude that introducing cangrelor for the treatment of patients undergoing PCI falls within a reasonable economic margin in each of the countries.

Although the absolute reductions predicted in our analysis (314 hospital days and 190 clinical events avoided over 5 years) may appear modest in the context of more than 54,000 STEMI patients, these findings remain clinically meaningful. Even small relative improvements translate into the prevention of serious events such as MI, ST, and death, which carry considerable implications for patient outcomes. Reductions in hospital days, even if limited in absolute terms, are valuable within an already resource-constrained NHS setting. Taken together, these results reinforce the potential system-level and clinical importance of introducing cangrelor in this patient population.

Some of the alternative scenarios explored in the BIM, such as assuming a complete lack of absorption of oral P2Y12 inhibitors, represent extreme conditions. These assumptions were included to test the boundaries of potential outcomes and should not be interpreted as reflective of routine clinical practice. As such, the larger predicted benefits of cangrelor in these scenarios are best considered as upper-bound estimates rather than expected real-world effects.

There are limitations in the present BIM. Firstly, the BIM uses data from the pivotal CHAMPION POOLED trials, conducted over ten years ago, on which the 2015 European Medicines Agency (EMA) marketing authorization is based [11]. Whilst clinical practice has changed in recent years, newer non-randomised studies have further reported the efficacy of cangrelor [27,49,50,51,52,53]. The inputs used in the analysis were also taken from published clinical trials, meta-analyses, or real-world evidence, and validated by a UK clinical expert, some assumptions were necessary and therefore some uncertainty may remain. For example, the reduction in LOS was sourced from a single-centre US study in a general PCI population, and not specifically in STEMI patients with gastric absorption issues [25]. However, the applicability of this US-based study to a UK population was validated by a consultant interventional cardiologist who agreed that bleeding events lead to a longer LOS in hospital.

Bailout GPI usage assumptions in the model (10%) were conservative, given that GPI usage in BCIS 2022–2023 was 32.6% in patients undergoing primary PCI (PPCI) in the UK [18]. Clinical expert opinion concludes that only a small proportion of GPI usage is upfront in STEMI patients with gastric absorption issues undergoing PCI, given that GPIs are primarily referenced as a bailout treatment option in the 2023 ESC Guidelines for the management of acute coronary syndromes [6]. Finally, an average patient weight of 70 kg was assumed in the model despite STEMI patients potentially weighing more than average. The most appropriate average weight of STEMI patients is unclear. However, a range of cangrelor/GPI dosing and cost was explored through OWSA. It should be noted that increasing the weight assumption to 95 kg, informed by the upper quartile baseline value reported in the White et al., 2012 CHAMPION-POOLED publication, had no impact on the number of cangrelor vials needed for a patient [54].

A further limitation is that certain scenario analyses (e.g., assuming complete lack of oral P2Y12 absorption) may overstate the benefits of cangrelor. These scenarios were intentionally modelled to test worst-case conditions, and results should be interpreted with caution.

Despite these limitations, this budget impact analysis robustly demonstrates that the use of cangrelor in STEMI patients with gastric absorption issues is associated with small cost saving to the NHS over 5 years. The pharmacologic properties of cangrelor mean it is not only an attractive agent for protection of ischemic events in patients undergoing PCI, but also a predictable option in case of procedural complications, such as bleeding or need for emergent surgery, given its fast offset of effects, obviating the need for an antidote for reversal which is not formally accounted for in this cost analysis [55]. The benefit of cangrelor for reducing the risk of in-hospital bleeding cannot be ignored, given recent evidence highlighting a significant association between increased mortality, major bleeding and reinfarction with in-hospital bleeding at the 1-year follow up [56].

Further emphasizing the unmet need, some eptifibatide products have recently been discontinued, owing to a shortage of the active ingredient, emphasizing the need for more treatment options for STEMI patients with gastric absorption issues undergoing PCI. The shortage of eptifibatide has led to an increase in the price per 75 mg/100 mL vial from GBP 42.79 in 2023 to GBP 299 in 2024 [41,42,57,58].

## 5. Conclusions

Cangrelor represents an effective treatment option for STEMI patients with gastric absorption issues undergoing PCI. A minimal budget would be required to incorporate cangrelor into this population in the UK and may result in a small cost saving driven by reduced bailout GPI usage and reduced LOS in hospitals for some patients.

## Figures and Tables

**Table 1:** Current market share and market uptake of antiplatelet therapies.

		Cangrelor	Clopidogrel	Prasugrel	Ticagrelor	Eptifibatide	Tirofiban	Aspirin and Heparin Alone
Current scenario ^a^		0%	10%	50%	30%	2.5%	2.5%	5%
Future scenario ^b^	Year 1	10%	9%	45%	27%	2.25%	2.25%	4.5%
	Year 2	15%	8.5%	42.5%	24%	2%	2%	4%
	Year 3	20%	8%	40%	21%	1.75%	1.75%	3.5%
	Year 4	25%	7.5%	37.5%	18%	1.5%	1.5%	3%
	Year 5	30%	7%	35%	15%	1.25%	1.25%	2.5%

Notes: ^a^ Market share estimates for existing antiplatelet treatments were guided by clinical expert input. ^b^ Clinical expert opinion was used to validate expected uptake of cangrelor.

**Table 2:** Safety and efficacy data used in the budget impact model.

	Efficacy at 48 h of PCI Shown by % Rates of Each Event				Safety Outcomes, TIMI, at 48 h of PCI		Reference
	ST	MI	IDR	Death	Major	Minor	
Cangrelor	0.10%	2.06%	0.29%	0.20%	0.20%	0.49%	Westman et al., 2017 [22]
Clopidogrel	0.85%	3.65%	0.74%	0.36%	0.22%	0.41%	Steg et al., 1992 [24]
Prasugrel	0.47%	2.74%	0.55%	0.19%	0.20%	0.44%	Westman et al., 2017 [22]
Ticagrelor	0.53%	2.66%	0.64%	0.36%	0.21%	0.65%	Westman et al., 2017 [22]
Eptifibatide	0.59%	2.45%	0.78%	0.39%	0.78%	1.57%	Vaduganathan et al., 2017 [23]
Tirofiban	0.59%	2.45%	0.78%	0.39%	0.78%	1.57%	2.5%
Aspirin & heparin ^a^	0.85%	3.65%	0.74%	0.36%	0.22%	0.41%	Steg et al., 1992 [24]

Notes: ^a^ Efficacy assumed to be equal to clopidogrel due to lack of data—a conservative assumption that favours aspirin and heparin alone. Abbreviations: IDR, ischemia-driven revascularization; MI, myocardial infarction; PCI, percutaneous coronary intervention; ST, stent thrombosis; TIMI, thrombolysis in myocardial infarction.

**Table 3:** Cost data used in the model.

Event	Cost Per Event	Reference
Ischemic events ^a^		
ST	GBP 309.06	Danese et al., 2016 [31]
MI	GBP 309.06	Danese et al., 2016 [31]
IDR	GBP 309.06	Danese et al., 2016 [31]
Bleeding events ^b^		
Major	GBP 381.74	Mamas et al., 2018 [32]
Minor	GBP 381.74	Mamas et al., 2018 [32]
Cardiac death	GBP 0.00	Lizano-Diez et al. [33]
Antiplatelet therapy ^c^		
Cangrelor	GBP 250.00	BNF [34]
Clopidogrel	GBP 0.16	BNF [35], SmPC [36]
Prasugrel	GBP 0.88	BNF [37], SmPC [38]
Ticagrelor	GBP 1.95	BNF [39], SmPC [40]
Eptifibatide ^d^	GBP 897.00	BNF [41], SmPC [42]
Tirofiban ^d^	GBP 292.22	BNF [43], SmPC [44]
Aspirin and heparin alone ^e^	GBP 0.00	
Cangrelor	GBP 250.00	BNF [34]
Hospital stay cost		
Post-operative day in hospital	GBP 498.50	NHS reference costs [45]

Notes: ^a^ All costs assumed to be equal to the cost of myocardial infarction based on the approach by Lizano-Díez and Paz Ruiz [42]. Hospitalization accounted for 95% of ischemic event costs [31]. ^b^ Major and minor bleeding costs assumed to be equal based on the approach by Lizano-Díez and Paz Ruiz [33]. ^c^ All drug costs calculated for an average 70 kg adult. There are no additional administration costs. ^d^ The duration of infusion is assumed to be 24 hours based on the summary of product characteristics (SmPC) [42,44]. ^e^ Aspirin and heparin costs are excluded as assumed to be equal in all treatment arms. Split between eptifibatide and tirofiban for bailout GPI use is assumed to be equal. Bailout GPI dose is assumed to be equal to routine GPI dose. Abbreviations: GPI, glycoprotein inhibitor; IDR, ischemia-driven revascularization; MI, myocardial infarction; ST, stent thrombosis.

**Table 4:** Base case budget impact summary results.

Parameter	Total over 5 Years	Average over 5 Years
Patients treated with cangrelor, N	10,903	2181
Total costs in scenario without cangrelor, GBP	74,948,153	14,989,631
Total costs in scenario with cangrelor, GBP	74,730,151	14,946,030
Net budget impact, GBP	−218,002	−43,600
Budget impact, %	−0.29	−0.29

**Table 5:** One-way sensitivity analysis results.

Parameter	Base Case Value	−20%	+20%	Lower Bound BI	Upper Bound BI	Difference Between Upper and Lower Bound BI Result
Bailout GPI use—current scenario	0.10	0.06	0.14	GBP 868,552	−GBP 1,537,205	GBP 2,405,756
Cost of bailout GPIs—current scenario	59.461	38.48	84.93	GBP 868,552	−GBP 1,537,205	GBP 2,405,756
Cost of cangrelor	250	161.79	357.10	−GBP 1,179,760	GBP 949,682	GBP 2,129,442
GPI—additional days in hospital	3.10	2.01	4.43	GBP 79,248	−GBP 578,898	GBP 658,146
Other comparators—additional days in hospital	2.52	1.63	3.60	−GBP 459,950	GBP 75,750	GBP 535,700
Bailout GPI use—future scenario	0.02	0.01	0.03	−GBP 459,561	GBP 75,277	GBP 534,838
Cost of bailout GPIs—future scenario	13.081	8.466	18.69	−GBP 459,561	GBP 75,277	GBP 534,838
Patients undergoing PCI	GBP 94,103.00	GBP 60,898.50	GBP 134,417.09	−GBP 141,080	−GBP 311,396	GBP 170,316
Number of patients eligible for treatment with cangrelor	GBP 10,902.65	GBP 7,055.62	GBP 15,573.39	−GBP 141,080	−GBP 311,396	GBP 170,316
Patients suitable for cangrelor	0.12	0.07	0.16	−GBP 140,067	−GBP 310,339	GBP 170,271

Abbreviations: BI, budget impact; GPI, glycoprotein inhibitor; PCI, percutaneous coronary intervention.

## Data Availability

The original contributions presented in this study are included in the article. Further inquiries can be directed to the corresponding author(s).

## Abbreviations

The following abbreviations are used in this manuscript:

ADP	Adenosine diphosphate
BCIS	British Cardiovascular Intervention Society
BI	Budget impact
BIM	Budget impact model
BNF	British National Formulary
CVD	Cardiovascular diseases
EMA	European Medicines Agency
ESC	European Society of Cardiology
GPI	Glycoprotein IIb/IIIa receptor inhibitors
HES	Hospital episode statistics
IDR	Ischemia-driven revascularisation
ISPOR	The Profession Society for Health Economics and Outcomes Research
IV	Intravenous
LOS	Length of stay
MI	Myocardial infarction
NHS	National Health Service
NICE	National Institute of Clinical Excellence
OHCA	Out-of-hospital cardiac arrest
OWSA	One-way sensitivity analysis
PCI	Percutaneous coronary intervention
PPCI	Primary percutaneous coronary intervention
PSS	Personal Social Services
ST	Stent thrombosis
STEMI	ST-elevation myocardial infarction
TIMI	Thrombolysis in myocardial infarction
